# Active and latent tuberculosis in refugees and asylum seekers: a systematic review and meta-analysis

**DOI:** 10.1186/s12889-020-08907-y

**Published:** 2020-06-03

**Authors:** Raquel Proença, Fernanda Mattos Souza, Mayara Lisboa Bastos, Rosângela Caetano, José Ueleres Braga, Eduardo Faerstein, Anete Trajman

**Affiliations:** 1 State University of Rio de Janeiro, Rio de Janeiro, RJ Brazil; 2 Oswaldo Cruz Foundation, Rio de Janeiro, RJ Brazil; 3 McGill University, Montreal, QC Canada; 4 Federal University of Rio de Janeiro, Rio de Janeiro, RJ Brazil

**Keywords:** Forced migration, *Mycobacterium tuberculosis*, Latent tuberculosis infection, Prevalence, Global health

## Abstract

**Background:**

In 2018, there were 70.8 million refugees, asylum seekers and persons displaced by wars and conflicts worldwide. Many of these individuals face a high risk for tuberculosis in their country of origin, which may be accentuated by adverse conditions endured during their journey. We summarised the prevalence of active and latent tuberculosis infection in refugees and asylum seekers through a systematic literature review and meta-analyses by country of origin and host continent.

**Methods:**

Articles published in Medline, EMBASE, Web of Science and LILACS from January 2000 to August 2017 were searched for, without language restriction. Two independent authors performed the study selection, data extraction and quality assessment. Random effect models were used to estimate average measures of active and latent tuberculosis prevalence. Sub-group meta-analyses were performed according to country of origin and host continent.

**Results:**

Sixty-seven out of 767 identified articles were included, of which 16 entered the meta-analyses. Average prevalence of active and latent tuberculosis was 1331 per 100 thousand inhabitants [95% confidence interval (CI) = 542–2384] and 37% (95% CI = 23–52%), respectively, both with high level of heterogeneity (variation in estimative attributable to heterogeneity [I^2^] = 98.2 and 99.8%). Prevalence varied more according to countries of origin than host continent. Ninety-one per cent of studies reported routine screening of recently arrived immigrants in the host country; two-thirds confirmed tuberculosis bacteriologically. Many studies failed to provide relevant information.

**Conclusion:**

Tuberculosis is a major health problem among refugees and asylum seekers and should be given special attention in any host continent. To protect this vulnerable population, ensuring access to healthcare for early detection for prevention and treatment of the disease is essential.

## Background

In 2018, there were 70.8 million refugees, asylum seekers and displaced persons worldwide, the largest number ever recorded [1]. A refugee is someone who “owing to well-founded fear of being persecuted for reasons of race, religion, nationality, membership of a particular social group or political opinion, is outside the country of his nationality and is unable or, owing to such fear, is unwilling to avail himself of the protection of that country” [2]. Asylum seekers are persons who claim to be admitted to a country as refugees and are awaiting the authorities’ decision [3].

Refugees and asylum seekers may have a significant burden of infectious diseases, such as tuberculosis, malaria, viral hepatitis and parasitic infections, as a result of the prevalence of such diseases in their country of origin and of exposure to adverse conditions during migration and after arrival at the host country [4–7]. They usually come from countries where different communicable diseases are endemic and often received minimal medical care prior to departure [8, 9]. In addition, confinement for years in conditions of overcrowding and insalubrity in shelters, rural camps or urban slums make them highly vulnerable to communicable diseases [10–12].

Tuberculosis is a major cause of human mortality globally [13]. *Mycobacterium tuberculosis* infects 23% of the global population, [14] and in the absence of treatment for latent *M. tuberculosis* infection (LTBI), 5 to 10% of these individuals can develop active tuberculosis, most within 2 years of infection [15]. Risk of progression from LTBI to active disease among migrants is higher throughout their journey and may last longer after arrival in host countries [16, 17]. Effective treatment of LTBI can reduce up to 90% the risk of progression to active tuberculosis, and is considered now a major action to eliminate the disease by 2050, as proposed by the End Tuberculosis Strategy [18].

Refugees, asylum seekers and internally displaced migrants live in heterogeneous socio-economic conditions and have various origins, reasons for fleeing and legal status. Yet, overall, compared with other categories of immigrants, they may be at higher risk for tuberculosis either having arrived with active tuberculosis in the destination country, or from developing active tuberculosis from previous LTBI or from acquiring the disease upon arrival [19, 20]. The debatable “healthy migrant effect” may not apply to this highly vulnerable population [21].

A previously published systematic review [20] has analysed the prevalence of tuberculosis among all immigrants and summarised data from 1980 to 2004, before the more recent migratory crisis. Additionally, a narrative review on infectious diseases in refugees was published, with data on active and latent tuberculosis from 29 articles from 2010 to 2016 [22]. Other reviews have also been published on refugees in specific scenarios, such as the effectiveness and coverage of tuberculosis screening in Europe [23, 24], tuberculosis in refugee camps [17], yield of screening for active tuberculosis in Germany [25], and prevalence of tuberculosis in the United Kingdom [26]. To our knowledge, no systematic review on active and latent tuberculosis prevalence in refugees is available. The current study aimed to summarise the prevalence of LTBI and active tuberculosis in refugees and asylum seekers, despite their high heterogeneity as a population.

## Methods

### Search strategy

We searched the bibliographic databases MEDLINE, EMBASE, LILACS and Web of Science, using the terms “tuberculosis”, “prevalence”, “refugee”, “asylum seekers”, “forced migration”, as MesH terms and text word. Strategy searches are available in the supplement material (Table S1 and S2).

The search was conducted in August 2017, without language or other restrictions. Studies published between January 2000 and August 2017 were eligible in order to contemplate the recent immigration crisis. The cut-off for the initial date was based on the trend of numbers of manuscripts published (Figure S1). We also searched the lists of references of the included studies, reviews and government reports.

### Study selection

The study selection, data extraction and quality assessment of studies were carried out by two independent reviewers (RP and FMS). Disagreements were solved by consensus or by two other reviewers (AT and MB). In addition, a 10% sample of the excluded studies was examined by reviewers AT and MB.

Reference data were stored in the EndNote web reference manager [Thomson Reuters (SCIENTIFIC), NYC, USA], and duplicate references were discarded. The selection was performed in two steps: screening of titles and abstracts, and full text evaluation. Although the search did not restrict language, only studies wrtitten in English, French, Spanish or Portuguese were included in the following steps. All studies on active tuberculosis or LTBI in the targeted population were included if the estimation of prevalence was reported or data were available for its calculation. Studies including mixed populations, i.e., not exclusively refugees and asylum seekers were also included if prevalence could be extracted by stratum

There were no restrictions on the tuberculosis clinical characteristics (pulmonary or extrapulmonary, drug susceptible or resistant) or study population (as to sex, age or country of origin and host continent). Cross-sectional, cohort studies and clinical trials were eligible. We restricted the selection to studies with at least 30 individuals.

For the diagnosis of active tuberculosis, smear microscopy, culture or molecular tests (Xpert® MTB/RIF and others), as well as clinical and radiological criteria, were accepted. For the diagnosis of LTBI, tuberculin skin testing (TST) or interferon-gamma release assays (IGRA) were accepted, and the presence of LTBI was considered if any of the two tests was positive [27]. We followed TST cut-off points for LTBI definition used by the study authors.

### Data collection process

Data extraction was conducted using an electronic form built on the EpiData 3.1 software (Epidata Association, Odense, Denmark). Whenever available, information on the number of individuals, events of interest and prevalence rates was collected by country of origin and host continent, to perform subgroup analyses. Studies with inpatients were classified as “hospitalised populations”. If the study was carried out in a hospital with outpatients, participants were not classified as hospitalised.

### Methodological quality of studies

Quality assessment of studies was carried out by two independent reviewers (RP and FMS) based on the document “Strengthening the Reporting of Observational Studies in Epidemiology” (STROBE) [28]. Differences were resolved by consensus. High quality was defined as at least 80% of STROBE criteria fulfilled, average quality as 50–79% of criteria were fulfilled and low quality as less than 50% of criteria fulfilled. Additionally, non-bacteriologically confirmed diagnosis of tuberculosis was considered to increase the risk of information bias, and non-routine screening was considered to increase the risk of selection bias. All studies that reported the necessary information (i.e., country of origin or host continent) were included in the meta-analyses, regardless of their quality.

### Data analyses

Study characteristics, population profile, setting and methodological aspects were described using frequency tables. Refugees and asylum seekers constitute a highly heterogeneous group of people, depending not only on individual cultural and socio-economic characteristics, but also on the reasons for fleeing their country and their legal status in the host country. We hypothesised that origin and destination could influence the prevalence of active tuberculosis and LTBI and thus opted to perform a meta-analysis by country of origin and a subgroup analysis by continent of destination. For these analyses, we used the studies that contained this information. Average prevalence rates and their 95% confidence intervals (CI) were estimated using a random effect model. Freeman-Tukey transformation was used to stabilise variance measures. Heterogeneity analysis was performed using variation in estimative attributable to heterogeneity (I^2^) statistics and Q chi-square test. All statistical analysis and Forest plots were performed using the STATA 13 software (module metaprop) (StataCorp LP, College Station, USA) [29].

The Preferred Reporting Items for Systematic Reviews and Meta-Analyses (PRISMA) [30] was used for reporting. A filled PRISMA form for this manuscript is available in the supplementary material (Chart S1). The full review protocol is available in PROSPERO, registration number CDR42016052361.

## Results

We identified 767 references, of which 282 were duplicated and thus excluded. After reading the titles and abstracts of the 485 studies, 170 were selected for full text reading. For abstract selection, initial agreement between the two main reviewers was 88%. In the 10% sample selected for check by the two additional reviewers, initial agreement was 93%. For full texts, initial agreement between the two main reviewers was 85%. In the 10% sample checked by the two additional reviewers, initial agreement was 80%. Final consensus was obtained in 100% of them.

**Fig. 1:**
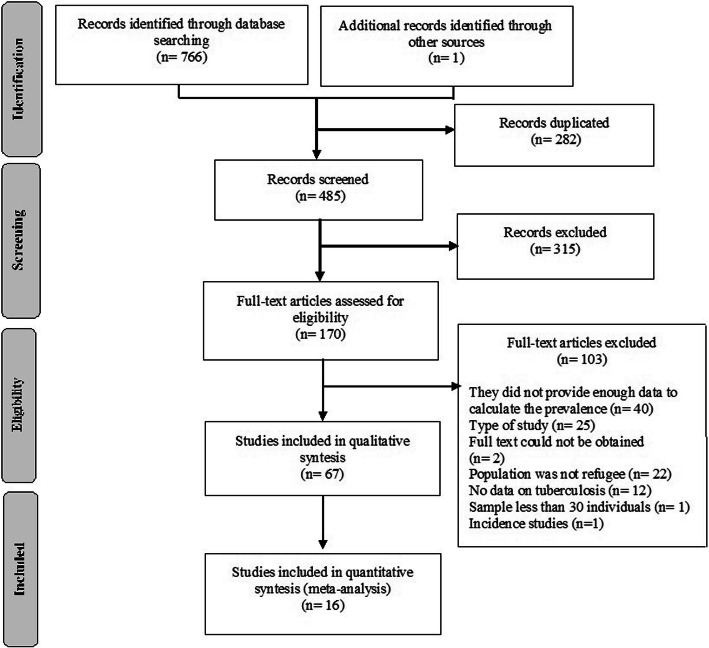
Flowchart showing inclusions and exclusions from the systematic review

Sixty-seven studies were included in the present review. Reasons for exclusion of the other 103 studies are displayed in Fig. 1. Information on countries of origin and host continent was available in 16 studies, which were included in the meta-analyses.

Sixty-six studies were published in English and one in French [31]. Fifteen studies reported active tuberculosis prevalence, 21 reported LTBI prevalence and 31 reported both. The total screened population was 599,072.

### Active tuberculosis prevalence

#### Study characteristics and population

Among the 46 studies that reported active tuberculosis prevalence, 56.5% were cross-sectional; none were clinical trials (Table 1). Two thirds of these studies were published after 2009, and among them, half completed data collection before 2011. Sixty-three per cent of studies included over 500 people.

**Table 1:** Studies on prevalence of active tuberculosis included in the systematic review and their characteristics (*n* = 46)

First Author	Year of publication	Country of study	Population	Country / Region of origin	Period of data collection		Setting	Sample size (n)	Study design	Women (%)	Bacteriologically confirmed	Prevalence of Active TB (per 100,000 inhabitants)		
					Start	End						Men	Women	Overall
Mockenhaupt [32]	2016	Germany	Refugee	Syria	2011	2015	Routine screening	44 ^b^	Cohort	34.1	NI	NI	NI	11,364
Nisbet [4]	2007	New Zealand	Refugee	Ethiopia; Somalia; Vietnam	1993	2004	Routine screening	98 ^b^	Cohort	44.0	NI	NI	NI	10,204
Ravensbergen [33]	2016	Netherlands	Asylum Seeker	Eritrea; Syria; Afghanistan; Armenia; Nigeria	2014	2015	Routine screening	736 ^a, b^	Cross sectional	32.6	Yes	NI	NI	6250
Rajamanoharan [34]	2004	UK	Mixed	Africa; Eastern Europe; Middle East; South America; Asia	2001	2002	NI	1856 ^b^	Cross sectional	NI	NI	NI	NI	6627
Marras [35]	2003	Canada	Refugee	India; Tibet; Nepal	1998	2001	Routine screening	525 ^b^	Cohort	32.0	Yes	NI	NI	5905
Lee [36]	2013	USA	Refugee	Iraq; Burma; Bhutan; Iran; Cuba; Thailand; Vietnam; Nepal; Somalia	2009	2009	Routine screening	78,899 ^b^	Cross sectional	NI	Yes	NI	NI	4349
Lobue [37]	2004	USA	Refugee	NI	2001	2003	Routine screening	54 ^b^	Cohort	NI	Yes	NI	NI	3704
Gray [38]	2012	Australia	Refugee	Middle East; Africa; Asia; Australia	2005	2010	Routine screening	328 ^b^	Cross sectional	45.7	No	NI	NI	3354
Banfield [39]	2012	Australia	Refugee	Sudan; Congo; Liberia; Burundi; Tanzania; Sierra Leone; Burma	2006	2007	Routine screening	264 ^c^	Cross sectional	51.2	No	NI	NI	3030
Otoukesh [40]	2012	Iran	Refugee	Afghanistan	2005	2010	Routine screening	23,152 ^b^	Cross sectional	52.3	NI	2.01	3.16	2613
Dierberg [41]	2016	India	Refugee	Tibet	2011	2013	NI	3830 ^c^	Cohort	35.7	Yes	3.12	1.39	2507
Tiong [42]	2006	Australia	Refugee	Western Africa; Central Africa; Eastern Africa; Africa	2004	2005	Both	84 ^c^	Cross sectional	55.8	No	NI	NI	2381
Chaves [43]	2009	Australia	Refugee	Myanmar; Thailand	2004	2008	Symptomatic	149 ^a, c^	Cohort	48.7	Yes	NI	NI	2013
Sheikh [44]	2009	Australia	Refugee	Asia; Middle East; Western Africa; Central Africa; Eastern Africa	2005	2006	Routine screening	219 ^c^	Cross sectional	53.1	No	NI	NI	1826
Lowther [45]	2012	USA	Refugee	Sub-Saharan Africa; South and Southeast Asia	2000	2007	Routine screening	11,615^c^	Cohort	44.8	Yes	NI	NI	1817
Oeltmann [46]	2008	USA	Refugee	Thailand	2004	2005	Routine screening	15,455^b^	Cross sectional	41.2	Yes	NI	NI	1760
Sarivalasis [47]	2012	Switzerland	Asylum Seeker	Balkan Peninsula; Soviet Union; Asia; Africa	2009	2010	Routine screening	393^c^	Cross sectional	27.2	Yes	1.40	0.93	1272
Harstad [48]	2010	Norway	Asylum Seeker	Iraq; Somalia; Russia; Afghanistan; Serbia and Montenegro	2005	2008	Routine screening	2237^b^	Cohort	30.1	Yes	0.83	2.23	1252
Varkey [49]	2007	USA	Refugee	Africa; Europe; Asia; Central and South America	1997	2001	Routine screening	9842^c^	Cohort	47.7	Yes	1.13	0.65	1179
Choi [6]	2007	Republic of Korea	Refugee	North Korea	1999	2006	Routine screening	7722 ^b^	Cross sectional	63.4	Yes	1.34	1.00	1127
Soydan [50]	2017	Turkey	Mixed	NI	2013	2015	Symptomatic	1149^a, b^	Cross sectional	47,3	No	0.82	1.10	957
Bua [51]	2016	Italy	Mixed	Nigeria; Mali; Senegal; Bangladesh; Gambia; Guinea; Nigeria; Ghana; Togo	NI	NI	Routine screening	109^b^	Cross sectional	NI	Yes	NI	NI	917
Diel [52]	2004	Germany	Asylum Seeker	Afghanistan; Turkey; Iran; Burkina Faso; Socialist Federal Republic of Yugoslavia; Sierra Leone; Russia; Guinea; Togo	1997	2002	Both	12,176^b^	Cohort	NI	Yes	NI	NI	887
Bennet [53]	2017	Sweden	Asylum Seeker	Afghanistan; Somalia; Eritrea; Ethiopia	2015	2016	Routine screening	2936 ^b^	Cohort	NI	Yes	NI	NI	817
Tafuri [10]	2011	Italy	Asylum Seeker	Asia; Eritrea; Gambia; Ghana; Nigeria; North Africa; Somalia; Tunisia	2009	2009	Routine screening	982^c^	Cross sectional	14.4	Yes	NI	NI	815
Russo [54]	2016	Italy	Asylum Seeker	Africa; Asia; South America	2012	2013	Symptomatic	792^c^	Cross sectional	20,1	No	NI	NI	758
Lalchandani [55]	2001	Ireland	Refugee	Africa; Romania; Kosovo; Russia	1999	2000	Routine screening	271^a, b^	Cross sectional	100	NI	NI	NI	738
Johnston [56]	2012	Australia	Refugee	Asia; Africa	2009	2010	Routine screening	176 ^c^	Cohort	41.7	NI	NI	NI	568
Lim [57]	2016	Canada	Refugee	Tibet	2014	2016	Routine screening	180 ^b^	Cohort	51,1	Yes	NI	NI	556
Hobbs [58]	2002	New Zealand	Asylum Seeker	Iran; Afghanistan; Siri Lanka; Czech Republic; Kuwait; Somalia; Iraq	1999	2000	Routine screening	780 ^c^	Cross sectional	31.9	No	NI	NI	513
Lobato [59]	2008	USA	Refugee	Bosnia; Vietnam, Somalia; Congo; Eritrea; Haiti; Cambodia	1996	2005	Routine screening	4904 ^c^	Cohort	NI	Yes	NI	NI	510
Winje [60]	2008	Norway	Asylum Seeker	Asia. Europe; Africa	2005	2006	Routine screening	1000 ^b^	Cross sectional	24.9	Yes	NI	NI	500
Liu [61]	2015	USA	Refugee	NI	2007	2012	Routine screening	232,738 ^b^	Cross sectional	NI	Yes	NI	NI	474
Rennert-May [62]	2016	Canada	Refugee	Sub-Saharan Africa; Middle East; South Asia; East and Southeast Asia	2009	2011	Routine screening	746 ^c^	Cohort	50.1	Yes	NI	NI	402
Sane Schepisi [63]	2013	Italy	Mixed	NI	2009	2010	Routine screening	776 ^b^	Cross sectional	NI	Yes	NI	NI	258
Chai [64]	2013	USA	Mixed	Ethiopia; Cameroon; Eritrea; Sierra Leone; Togo; Vietnam; Sudan; Democratic Republic of Congo; Columbia	2003	2007	Routine screening	781 ^b^	Cohort	43.0	NI	NI	NI	256
Trauer [65]	2011	Australia	Refugee	Africa; Eastern Mediterranean; Southeast Asia	2006	2009	Routine screening	471 ^b^	Cohort	56.1	Yes	NI	NI	212
Gibson-Helm [66]	2015	Australia	Refugee	Siri Lanka; Afghanistan; India; Mauritius; Vietnam; Iraq; Indonesia; Pakistan; Philippines;	2002	2011	Routine screening	13,319 ^b^	Cross sectional	100.0	NI	NI	NI	188
Schneeberger Geisler [67]	2010	Switzerland	Asylum Seeker	NI	2004	2008	Routine screening	45,129 ^b^	Cross sectional	25.1	Yes	0.15	0.09	168
Harling [68]	2007	UK	Asylum Seeker	Iraq; Afghanistan; Czech Republic; Iran; Congo; Somalia; Zimbabwe; Angola	2002	2003	Routine screening	8258 ^b^	Cross sectional	25.0	Yes	NI	NI	133
Meier [69]	2016	Germany	Asylum Seeker	NI	2014	2015	Routine screening	11,773 ^b^	Cross sectional	NI	Yes	NI	NI	93
Bloch-Infanger [70]	2017	Switzerland	Asylum Seeker	NI	2004	2015	Routine screening	8697 ^b^	Cross sectional	NI	No	NI	NI	69
Weinrich [71]	2017	Germany	Refugee	NI	2015	2015	Routine screening	17,487 ^b^	Cross sectional	23,0	No	NI	NI	57
Yanni [72]	2013	Jordan	Refugee	Iraq	2007	2009	Routine screening	14,077 ^c^	Cohort	50.3	Yes	NI	NI	7
Subedi [73]	2015	USA	Refugee	Bhutan; Burma	2010	2012	Routine screening	149 ^b^	Cohort	46.3	Yes	NI	NI	0
Paxton [74]	2012	Australia	Refugee	NI	2006	2009	Routine screening	810 ^c^	Cross sectional	49.0	No	NI	NI	0

*Abbreviations*: *USA* United States of America, *UK* United Kingdom, *NI* Not Informed, *TB* Tuberculosis

In the column Setting, “both” means that the study performed routine screening and symptomatic evaluation

^a^Hospitalised population; ^b^Total population on study; ^c^Population screened for active tuberculosis

The total of individuals screened for active tuberculosis was 537,218, with a single study evaluating 232,738 individuals. Eighty-one per cent of studies were conducted in refugees (*n* = 437,264), 18% in asylum seekers (*n* = 95,283), and 0.9% in both (*n* = 4671).

The mean age, reported by 33% of studies [4, 10, 35, 39, 41, 50–52, 54, 55, 57, 62, 68, 69, 71], ranged from 18 to 40.7 years. Prevalence by sex was reported by only 17% of the studies.

The average time since arrival of refugees and asylum seekers to the host country to the time of evaluation for tuberculosis was 3.9 months (ranging from 0.7 to 12.8 months) in the 9% of studies [53, 64, 65, 73] conveying this information.

Sixty-seven (31/46) per cent of studies concomitantly evaluated the presence of LTBI in their populations. Among these, 57% used the LTBI diagnostic as a prerequisite to investigate the presence of active tuberculosis. In other words, they performed a diagnostic test for LTBI with TST or IGRA to rule out active tuberculosis; if they were positive, a chest X-ray was also performed and if it was indicative of active tuberculosis, bacteriological tests were conducted. Otherwise, TST or IGRA-positive individuals were considered to have LTBI.

#### Main findings

**Fig. 2:**
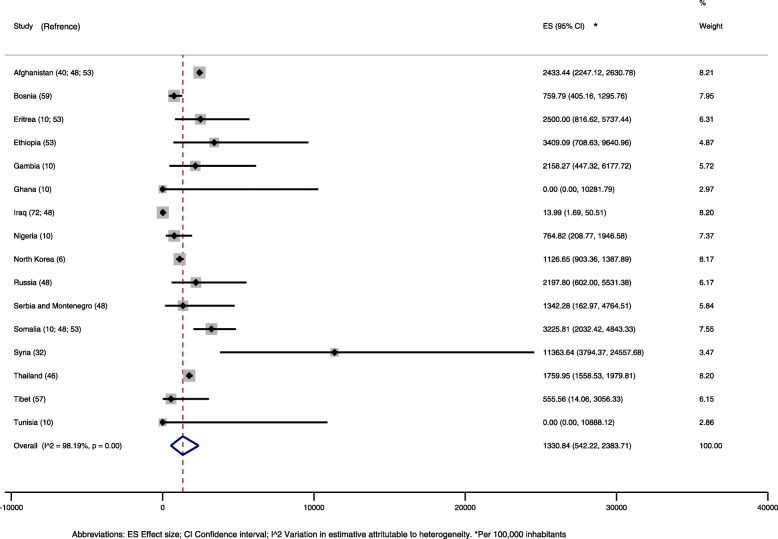
Prevalence of active tuberculosis in refugees and asylum seekers by country of origin

Active tuberculosis prevalence rates varied from 0 to 11,364 per 100 thousand inhabitants, with 89% of studies reporting values less than 5000 per 100 thousand. Considering studies that reported the prevalence by country of origin, the average prevalence of active tuberculosis was 1331/100 thousand inhabitants (95% CI, 542–2384), with high heterogeneity (I^2^ = 98%, Fig. 2). The prevalence was higher among refugees from Syria (11,364/100 thousand inhabitants, 95% CI, 3794–24,558), observed in a single study with 44 hospitalised participants. Studies with individuals from Ethiopia, Ghana and Tunisia had results with large confidence intervals, in samples smaller than 100 persons [10, 53].

**Fig. 3:**
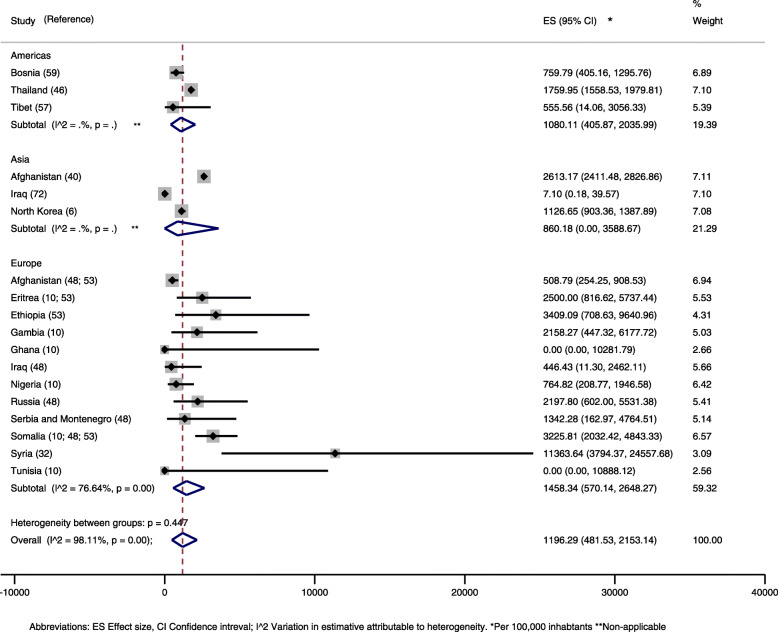
Prevalence of active tuberculosis in refugees and asylum seekers by host continent and country of origin

With regards to the host continent, refugees who immigrated to Europe, Asia and America presented a similar average prevalence of 1458, 860 and 1080 per 100 thousand inhabitants, respectively (Fig. 3). Europe was the continent that received refugees from the most diverse nationalities, better allowing an assessment of heterogeneity. In the other continents, this individual evaluation was not possible due to the small numbers. Refugees from Eritrea, Ethiopia and Somalia immigrated to Europe [10, 53] in the three studies that contained this information, and had a slightly larger tuberculosis prevalence. More information about the meta-analyses data on active tuberculosis can be found on the supplement material (Table S3).

### LTBI prevalence

#### Study characteristics and population

Fifty-four per cent of the 52 studies that reported LTBI prevalence were cohort studies; there were no clinical trials (Table 2). Sixty per cent of studies were published after 2009, and among them half completed data collection before 2011; one study did not provide this information. Thirty-one studies included over 500 participants.

**Table 2:** Studies on prevalence of latent tuberculosis infection included in the systematic review and their main findings (*n* = 52)

First Author	Year of publication	Country of study	Population	Country / Region of origin	Period of data collection		Setting	Sample size (n)	Women (%)	Study design	Diagnostic test	TST cutoff point (mm)	Prevalence of LTBI (%)		
													Men	Women	Overall
					Start	End									
Choi [6]	2007	Republic of Korea	Refugee	North Korea	1999	2006	Routine screening	1112 ^c^	63.4	Cross sectional	TST	10	NI	NI	81.47
Chaves [43]	2009	Australia	Refugee	Myanmar; Thailand	2004	2008	Symptomatic	149^a,c^	48.7	Cohort	TST / IGRA	10	NI	NI	70.47
Diel [52]	2004	Germany	Asylum Seeker	Afghanistan; Turkey; Iran; Burkina Faso; Socialist Federal Republic of Yugoslavia; Sierra Leone; Russia; Guinea; Togo	1997	2002	Both	12,176^b^	NI	Cohort	TST	NI	NI	NI	62.00
Tafuri [10]	2011	Italy	Asylum Seeker	Asia; Eritrea; Gambia; Ghana; Nigeria; North Africa; Somalia; Tunisia	2009	2009	Routine screening	982^c^	14.4	Cross sectional	TST	10	63.1	46.2	60.69
Lowther [45]	2012	USA	Refugee	Sub-Saharan Africa; South and Southeast	2000	2007	Routine screening	11,615^c^	44.8	Cohort	TST	NI	NI	NI	60.55
Baker [75]	2009	USA	Refugee	Somalia; Ethiopia; Liberia; Eritrea; Ghana; Zimbabwe; Ukraine; Russia Federation; China	2006	2007	Routine screening	195^c^	65.6	Cohort	TST / IGRA	10	65.7	32.0	55.38
Winje [60]	2008	Norway	Asylum Seeker	Asia. Europe; Africa	2005	2006	Routine screening	912^c^	24.9	Cross sectional	TST / IGRA	6	NI	NI	53.95
Lim [57]	2016	Canada	Refugee	Tibet	2014	2016	Routine screening	163^c^	51.11	Cohort	TST / IGRA	NI	54.2	43.8	51.53
Kowatsch-Beyer [76]	2013	USA	Refugee	Africa; East Asia; Eastern Europe; South Asia; Latin America	2008	2008	Routine screening	224^b^	47.8	Cohort	TST	5	NI	NI	51.34
Varkey [49]	2007	USA	Refugee	Africa; Europe; Asia; Central and South America	1997	2001	Routine screening	9842^c^	47.7	Cohort	TST	10	57.2	45.2	50.70
Watts [77]	2017	USA	Mixed	Africa; America; Europe; Eastern Mediterranean; Southeast Asia; West Pacific	1995	2012	Symptomatic	6669^b^	47	Cross sectional	TST	10	54.2	45.7	50.21
Padovese [78]	2013	Malta	Asylum Seeker	Somalia; Eritrea; Ethiopia; Western Africa	2010	2011	Routine screening	500^b^	18.8	Cross sectional	TST / IGRA	10	NI	NI	49.60
Liu [79]	2009	USA	Refugee	Ukraine; Vietnam; Somalia; Bosnia; Sudan	1999	2005	Routine screening	9132^c^	40.2	Cohort	NI	NI	NI	NI	49.17
Harstad [80]	2009	Norway	Asylum Seeker	Iraq; Somalia; Russia; Afghanistan; Serbia and Montenegro	2005	2006	Routine screening	4526 ^b^	30.6	Cohort	TST / IGRA	6	NI	NI	47.61
Ouimet [31]	2008	Canada	Asylum Seeker	India; Pakistan; Guinea; Congo; Nigeria; Ivory Coast; Cameroon; Siri Lanka; Mexico; Zimbabwe	2000	2004	Symptomatic	231 ^c^	43.2	Cross sectional	TST	NI	NI	NI	45.89
Weinfurter [81]	2011	USA	Refugee	NI	2004	2006	Routine screening	594^c^	47.8	Cross sectional	TST / IGRA	5	NI	NI	45.62
Pottie [82]	2007	Canada	Refugee	Sub-Saharan Africa; North Africa; Middle East; Eastern Europe	2004	2005	Routine screening	112 ^b^	59.8	Cohort	TST	10	55.6	34.3	42.86
Moreno [83]	2006	USA	Mixed	Africa; Latin America; Asia; Europe	1998	2001	Routine screening	101 ^c^	43.2	Cohort	TST	NI	NI	NI	41.58
Bertelsen [84]	2016	USA	Asylum Seeker	Sub-Saharan Africa; Asia; Eastern Europe; Middle East; Latin America and Caribbean; Western Europe	2012	2014	Routine screening	160 ^c^	33.8	Cohort	TST / IGRA	NI	NI	NI	40.62
Chai [64]	2013	USA	Mixed	Ethiopia; Cameroon; Eritrea; Sierra Leone; Togo; Vietnam; Sudan; Democratic Republic of Congo; Columbia	2003	2007	Routine screening	611 ^c^	43.0	Cohort	TST	10	NI	NI	39.12
Bua [51]	2016	Italy	Mixed	Nigeria; Mali; Senegal; Bangladesh; Gambia; Guinea; Nigeria; Ghana; Togo	NI	NI	Routine screening	109 ^b^	NI	Cross sectional	TST / IGRA	5	NI	NI	38.53
Lobato [59]	2008	USA	Refugee	Bosnia; Vietnam; Somalia; Congo; Eritrea; Haiti; Cambodia	1996	2005	Routine screening	4904 ^c^	NI	Cohort	NI	NI	NI	NI	38.38
Subedi [73]	2015	USA	Refugee	Bhutan; Burma	2010	2012	Routine screening	149 ^b^	46.3	Cohort	TST / IGRA	10	NI	NI	38.26
Goldberg [85]	2004	USA	Refugee	Soviet Union; Yugoslavia; Somalia	1999	2000	Routine screening	2194 ^c^	NI	Cohort	TST	10	NI	NI	37.06
Hobbs [58]	2002	New Zealand	Asylum Seeker	Iran; Afghanistan; Siri Lanka; Czech Republic; Kuwait; Somalia; Iraq	1999	2000	Routine screening	869 ^c^	31.9	Cross sectional	TST	10	NI	NI	36.36
Rennert-May [62]	2016	Canada	Refugee	Sub-Saharan Africa; Middle East; South Asia; East and Southeast Asia	2009	2011	Routine screening	746 ^c^	50.1	Cohort	TST / IGRA	NI	NI	NI	35.52
Board [86]	2016	USA	Refugee	Southeast Asia; South Asia; Middle East; Sub-Saharan Africa	2010	2013	Routine screening	9860^b^	45.8	Cross sectional	TST / IGRA	10	36.2	29.2	32.95
Nisbet [4]	2007	New Zealand	Refugee	Ethiopia; Somalia; Vietnam	1993	2004	Routine screening	100^b^	44.0	Cohort	TST	5	NI	NI	32.00
Trauer [65]	2011	Australia	Refugee	Africa; Eastern Mediterranean; Southeast Asia	2006	2009	Routine screening	458 †	56.1	Cohort	TST	10	36.0	28.6	31.88
Hensel [87]	2016	USA	Refugee	Burma; Bhutan; Iraq; Somalia	2013	2014	Routine screening	694 ^c^	45.0	Cross sectional	IGRA	NA	33.6	29.1	31.84
Walters [88]	2016	USA	Mixed	Sub-Saharan Africa; Eastern Asia and Pacific; Middle East; Southeast Asia; Latin America and Caribbean	2009	2012	Symptomatic	2244^b^	44.4	Cohort	TST / IGRA	10	NI	NI	30.30
Oeltmann [46]	2008	USA	Refugee	Thailand	2004	2005	Routine screening	5637 ^c^	41.2	Cross sectional	TST	5	NI	NI	28.81
Banfield [39]	2012	Australia	Refugee	Sudan; Congo; Liberia; Burundi; Tanzania; Sierra Leone; Burma	2006	2007	Routine screening	1004 ^c^	51.2	Cross sectional	TST / IGRA	NI	NI	NI	26.29
Tiong [42]	2006	Australia	Refugee	Western Africa; Central Africa; Eastern Africa; Africa	2004	2005	Both	96 ^c^	55.8	Cross sectional	TST / IGRA	10	NI	NI	25.00
Gray [38]	2012	Australia	Refugee	Middle East; Africa; Asia; Australia	2005	2010	Routine screening	328 ^b^	45.7	Cross sectional	TST	10	NI	NI	24.70
Sarivalasis [47]	2012	Switzerland	Asylum Seeker	Balkan Peninsula; Soviet Union; Asia; Africa	2009	2010	Routine screening	393 ^c^	27.2	Cross sectional	IGRA	NA	NI	NI	23.66
Sheikh [44]	2009	Australia	Refugee	Asia; Middle East; Western Africa; Central Africa; Eastern Africa	2005	2006	Routine screening	219 ^c^	53.1	Cross sectional	TST	10	NI	NI	23.29
Bennett [89]	2014	USA	Refugee	Middle East, Asia; Sub-Saharan Africa	2010	2012	Routine screening	4280 ^b^	49.7	Cohort	TST / IGRA	10	23.4	15.8	21.40
Paxton [74]	2012	Australia	Refugee	NI	2006	2009	Routine screening	810 ^c^	49.0	Cohort	IGRA	NA	NI	NI	20.86
Lucas [90]	2010	Australia	Refugee	Africa; Asia	2007	2008	Routine screening	524 ^b^	51.9	Cohort	TST / IGRA	10	NI	NI	18.32
Johnston [56]	2012	Australia	Refugee	Asia; Africa	2009	2010	Routine screening	176 ^c^	41.7	Cohort	TST	10	NI	NI	18.18
Marras [35]	2003	Canada	Refugee	India; Tibet; Nepal	1998	2001	Routine screening	525 ^b^	32.0	Cohort	TST	10	NI	NI	16.0
Ramos [91]	2010	USA	Refugee	Iraq	2007	2009	Routine screening	4923 ^c^	48.5	Cross sectional	TST / IGRA	NI	NI	NI	14.06
Bennet [53]	2017	Sweden	Asylum Seeker	Afghanistan; Somalia; Eritrea; Ethiopia	2015	2016	Routine screening	2936 ^b^	NI	Cohort	TST / IGRA	10	NI	NI	11.34
Mockenhaupt [32]	2016	Germany	Refugee	Syria	2011	2015	Routine screening	44 ^b^	34.1	Cohort	NI	NI	NI	NI	9.09
Liu [61]	2015	USA	Refugee	NI	2007	2012	Routine screening	57,019 ^c^	NI	Cross sectional	TST / IGRA	10	NI	NI	7.93
Taylor [92]	2016	USA	Refugee	NI	2010	2010	Routine screening	13,395 ^b^	NI	Cohort	TST / IGRA	10	NI	NI	6.20
Pavlopoulou [93]	2017	Greece	Mixed	Afghanistan; Congo; Bangladesh; Pakistan; Iran; Lebanon; Sudan; Kenia; Somalia; Eritrea	2010	2013	Routine screening	162 ^b^	44.4	Cross sectional	TST / IGRA	10	NI	NI	4.94
Harling [68]	2007	UK	Asylum Seeker	Iraq; Afghanistan; Czech Republic; Iran; Congo; Somalia; Zimbabwe; Angola	2002	2003	Routine screening	4275 ^c^	25.0	Cross sectional	TST	NI	2.4	0.9	2.18
Lee [36]	2013	USA	Refugee	Iraq; Burma; Bhutan; Iran; Cuba; Thailand; Vietnam; Nepal; Somalia	2009	2009	Routine screening	78,899 ^b^	NI	Cross sectional	TST / IGRA	10	NI	NI	1.87
Yanni [72]	2013	Jordan	Refugee	Iraq	2007	2009	Routine screening	13,669 ^c^	50.3	Cohort	TST	10	NI	NI	1.84
Entzel [94]	2003	USA	Refugee	Cuba	1999	2000	Routine screening	241 ^c^	NI	Cross sectional	TST	NI	NI	NI	0.41

*Abbreviations LTBI* Latent Tuberculosis Infection, *USA* United States of America, *UK* United Kingdom, *NI* Not Informed, *TST* Tuberculin skin test, *IGRA* Interferon gamma release assay, *NA* Not applicable

In the column Setting, “both” means that the study performed routine screening and symptomatic evaluation

^a^Hospitalised population; ^b^Total population total study; ^c^Population screened for LTBI

A total of 271,544 individuals were screened for LTBI: 233,688 individuals were refugees (reported by 67% of studies) and 27,960 individuals were asylum seekers (reported by 21% of studies). The remaining were studies including both types of situations.

The mean age, reported by 33% of studies [4, 10, 31, 35, 39, 51, 52, 57, 62, 68, 77, 78, 83, 84, 88, 93, 94], ranged from 3.5 to 39 years, with the lowest prevalence in a study that included only children (mean age 3.5 years). Only 21% of studies reported the prevalence by gender.

The average time since arrival of refugees and asylum seekers to the host country at the time of evaluation for LTBI was 3.8 months (range: 0.7–12.8 months) among the 13% of studies that reported this information [53, 64, 65, 73, 75, 82, 91].

Eighty-nine per cent of studies performed TST and 77% of those reported the TST cut-off point used to define LTBI: 10 mm was used in 78% of studies. Some studies considered different cut-off points to different populations (children, human immunodeficiency virus (HIV)-infected or BCG-vaccinated individuals).

#### Main findings

Prevalence of LTBI ranged from 0.4 to 81.5%, with 61% of the studies reporting a prevalence rate higher than 30%.

**Fig. 4:**
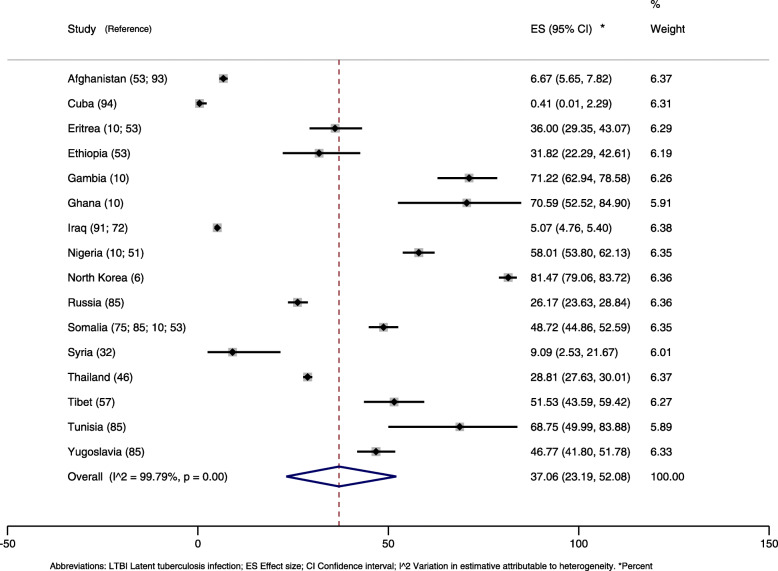
Prevalence of latent tuberculosis infection in refugees and asylum seekers by country of origin

In the meta-analysis by country of origin, prevalence rates were highly heterogeneous (I2 = 99.8%), with an average measure of 37% (95% CI, 23–52) (Fig. 4). Refugees from Cuba and Iraq presented the lowest rates, 0.4 and 5% respectively, and from North Korea, the highest rate, 81%, systematically screened when arriving in South Korea [6]. Targeted populations and sample sizes varied largely, with the Cuban study [94] evaluating 241 children under 7 years of age (and finding one LTBI case) and large systematic screening for active tuberculosis of Iraq refugees [72, 91] applying for visa or recently arrived in the United States of America (USA). Small sample sizes eventually resulted in very wide confidence intervals [10].

**Fig. 5:**
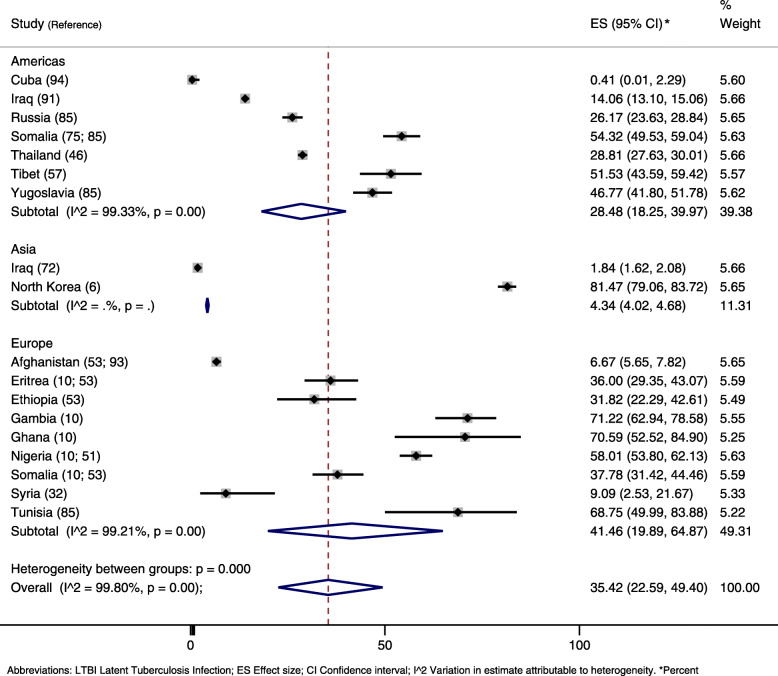
Prevalence of latent tuberculosis infection in refugees and asylum seekers by host continent and country of origin

In the subgroup analysis by host continent, refugees who immigrated to Europe presented the highest prevalence (41, 95% CI, 20–65), followed by those who went to the Americas (28, 95% CI, 18–40) (Fig. 5). However, one study in the USA excluded individuals with immunosuppressive conditions and thus had a high risk of false negative results [75]. Somali refugees who went to America had a higher prevalence rate (54%) than those who went to Europe (38%), while Iraq refugees who went to the Americas had a higher prevalence rate (14%) than those who went to Asia (2%). Overall, there were very few studies with information on country of origin and host continent. More information about the meta-analyses data on LTBI can be found on the supplement material (Table S4).

### Quality of studies and risk of bias

None of the 65 studies fulfilled all quality criteria. Among the 33 cross-sectional and the 32 cohort studies, only 13 and 11 respectively were considered high quality (Figure S2 and S3); 14 and 18 studies were of medium quality; and 6 cohort and 3 cross-sectional studies were considered low quality. Two studies [36, 91] were organization reports; it was not possible to perform the quality assessment.

In 85% of studies (*n* = 569,880), routine screening of all the individuals who arrived in the host country was the reason for the enrollment and 9% of studies tested individuals who sought health service with symptoms (*n* = 11,234). Only one study was conducted in refugee camps [46] (*n* = 15,455). Among the 37 studies that informed the diagnostic method for active tuberculosis, 73% confirmed tuberculosis bacteriologically.

Six per cent of studies involved hospitalised populations (*n* = 4). In these studies, overestimation of the prevalence is likely. Three studies [33, 50, 55] reported only active tuberculosis prevalence, and one described both active and latent tuberculosis [43]. None of them entered the meta-analyses.

Finally, only 15 articles used the 1951 United Nations Convention or a very similar definition of refugee and asylum seeker.

## Discussion

This systematic review and meta-analysis on tuberculosis prevalence in refugees identified 67 studies with a total of 599,072 evaluated individuals, of whom nearly half were evaluated for LTBI. The main finding was the high prevalence of active tuberculosis in these populations. Although some studies involving hospitalised populations may have overestimated the prevalence of the disease, most of the studies were conducted as routine screening in symptom-free individuals. The lower positive predictive value in symptom-free individuals may also result in overestimation of the prevalence, but despite these possible risks of bias, the prevalence is high.

Prevalence rates found in the current study, albeit very heterogeneous, were comparable to other very-high risk groups, such as prisoners and homeless [95, 96]. Although the highest prevalence rates were found in Syrians and among those who migrated to the Americas, these figures are based, respectively, on one and two studies solely. Furthermore, the results among Syrians refugees are from a highly selected setting and are based on a small sample: 5 among 44 hospitalised patients. Yet, this finding is worth highlighting: between 1990 and 2011 the tuberculosis prevalence in Syria had decreased from 85 to 23/100,000 [97]. Armed conflicts and wars destroy the basic medical infrastructure, undermine health agendas and cause significant shortages of health professionals and medicines, leading the prevalence of tuberculosis to a possible underestimation [8, 97]. Symptoms of the active phase of tuberculosis, such as coughing and fatigue, may go unnoticed to already infected individuals and health care workers in crisis settings, because they are insidious [17]. Dangerous situations encountered during migration, including overcrowding, incarceration, malnutrition, challenges to access health care, low adherence to treatment, associated risk of HIV infection and exposure to other migrants from higher incidence countries also contribute to the risk of contamination by *M. tuberculosis* and progression to disease [8, 97–99].

In regard to latent tuberculosis infection (LTBI), reported prevalence rates were also high, similar to those observed in populations characterised by high vulnerability to infection, such as prisoners [100], when compared to the overall population, in whom LTBI is expected to be 23% [14]. The exception was a study in children, an expected finding, as LTBI prevalence increases with age. LTBI prevalence was the highest among Somali refugees, in particular those who migrated to the Americas, in spite of possible underestimation due to exclusion of those with the highest probability of a false negative test result (immunosuppressed persons) in one of the two studies. The difference may reflect true heterogeneity in the populations migrating to these continents but could also reflect different methods of screening (TST cut-off, TST versus IGRA versus TST and IGRA) or targeted populations for screening in both continents. Alternatively, it could reflect differences in methodology regarding targeted population or testing criteria, but we did not find such differences. We cannot discard differences due to the choice of the continent of destiny or the journey itself, but this hypothesis remains speculative. Again, albeit based on few studies and the possible biases, the finding is noteworthy. Somalia is one of the poorest countries in the world and has also been facing a civil war in the Horn of Africa.

Despite the few studies per country of origin and per host continent, which limits the analysis according to these variables, and in spite of the heterogeneous populations involved, the high overall rates of active and latent tuberculosis found in the present review emphasise the responsibility of host countries to meet refugees’ specific health needs and of the global health community to fight tuberculosis in low-income countries from where most refugees flee, in order to attain WHO’s End Tuberculosis Strategy to eliminate the disease by 2050 [18]. In the host countries, there are still many challenges that need to be overcome for better care of refugees, such as lack of training of professionals, fear of breaches of confidentiality, fear of stigma and social rejection due to illness, fear of consequences in the immigration process due to the diagnosis of disease, insufficient information on the screening and treatment process, difficulty in communicating due to language differences, among others [23].

To the best of our knowledge, this is the first summarised analysis of tuberculosis among this specific subpopulation of migrants, and the first to include average measures according to their origin and destination. Among this review strengths is the reporting quality of most studies. In regard to bias, active tuberculosis was bacteriologically confirmed, and almost all derived from routine screening. These characteristics reduce the likelihood of overestimation.

Nonetheless, reported prevalence rates may be overestimated among symptomatic individuals in health facilities such as hospitals. Also, studies in populations applying for visa in countries with health restrictions may have underestimated prevalence of LTBI, since those known to be positive may give up application.

Moreover, this is a very heterogeneous group of individuals, and attempts to summarise any measure are challenging. The definition of “refugee” or “asylum seekers” was not clear in all studies. Age groups were highly heterogeneous as well, and prevalence of LTBI increases with age, thus influencing findings; in addition, language difficulties, fear of immigration authorities, lack of awareness of symptoms and fear of stigma may reduce the efficacy of tuberculosis detection mechanisms [8].

Origin and destination may reflect socio-economic status, reasons for fleeing, and tuberculosis setting, which explains our choice for meta-analyses. However, many studies could not be included in the meta-analyses due to lack of information about the country of origin. Thus, because only a few studies were eligible, all had to be included regardless of their quality. Some findings included in the meta-analyses refer to one or two studies only. Meta-regression could not be performed due to information gaps regarding study populations (e.g., gender, age, follow-up).

Additionally, most studies were performed in developed countries, and thus do not represent the majority of current refugees, who are hosted in low- and medium-income countries [101]. Generalizability and assertive conclusions are thus restricted. Lastly, although our bibliographic searches were finalised in August 2017, recent waves of forced migration are not entirely covered, because several studies refer to data collected up to 2011. More efforts and funds should be dedicated to international cooperation studies on tuberculosis – and other health issues - among forced migrants [102].

## Conclusion

Despite the highly heterogeneous prevalence across countries, active and latent tuberculosis seem to be frequent health issues among refugees and asylum seekers. Rapid screening is necessary in order to allow early detection and prompt treatment - or prevention - of the disease. This policy should aim at their protection against the disease, rather than their exclusion and discrimination. Efforts to guarantee their right to adequate health care cannot be overemphasised.

## Supplementary information

**Additional file 1: Figure S1.** Number of publications on Medline from 1945 to 2015 using descriptor “Tuberculosis AND Refugee”

**Additional file 2: Figure S2.** Evaluation of the quality of reporting of cohort studies according to STROBE criteria

**Additional file 3: Figure S3.** Evaluation of the quality of reporting of cross-sectional studies according to STROBE criteria

**Additional file 4: Table S1.** Key words used according to the bibliographic databases. **Table S2.** Structured search strategy according to PICOS axiom and bibliographic databases. **Table S3.** Studies included in the metanalyses for prevalence active tuberculosis (*n* = 10). **Table S4.** Studies included in the metanalyses for prevalence of latent tuberculosis infection (*n* = 13). **Chart S1.** Prisma Checklist

## Data Availability

All data generated or analysed during this study are included in this published article [and its supplementary information files].

## Abbreviations

CI: Confidence intervals
HIV: Human immunodeficiency virus
IGRA: Interferon-gamma release assays
LTBI: Latent tuberculosis infection
PRISMA: Preferred Reporting Items for Systematic Reviews and Meta-Analyses
STROBE: Strengthening the Reporting of Observational Studies in Epidemiology
TST: Tuberculin skin testing
I^2^: Variation in estimative attributable to heterogeneity
