# Supplementary figures and images for: Active and latent tuberculosis in refugees and asylum seekers: a systematic review and meta-analysis
Source: BMC Public Health. 2020 Jun 3;20:838. doi: 10.1186/s12889-020-08907-y (PMC7268459; doi:10.1186/s12889-020-08907-y)

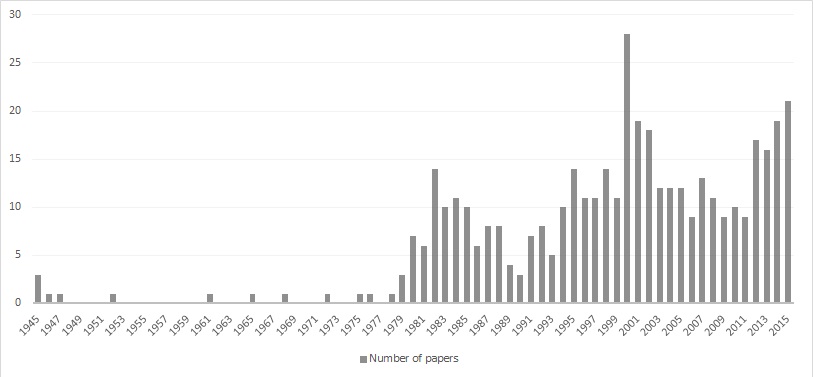

Supplement: Supplementary file 1 — Additional file 1: Figure S1. Number of publications on Medline from 1945 to 2015 using descriptor “Tuberculosis AND Refugee” [file 12889_2020_8907_MOESM1_ESM.jpg]

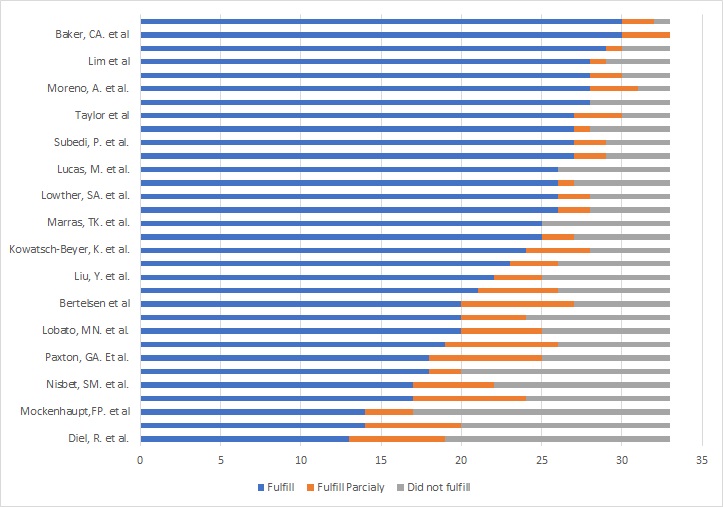

Supplement: Supplementary file 2 — Additional file 2: Figure S2. Evaluation of the quality of reporting of cohort studies according to STROBE criteria [file 12889_2020_8907_MOESM2_ESM.jpg]

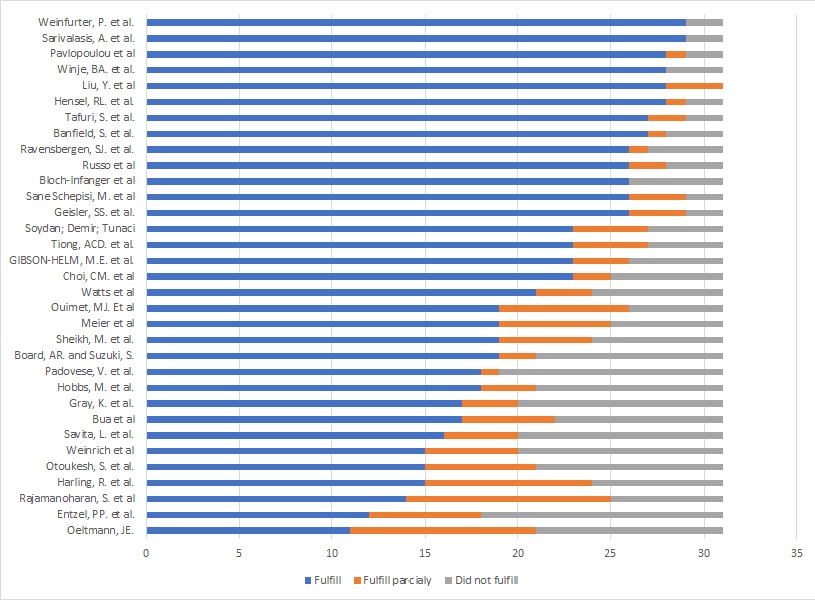

Supplement: Supplementary file 3 — Additional file 3: Figure S3. Evaluation of the quality of reporting of cross-sectional studies according to STROBE criteria [file 12889_2020_8907_MOESM3_ESM.jpg]
