# Supplementary material for: Active and latent tuberculosis in refugees and asylum seekers: a systematic review and meta-analysis
Source: BMC Public Health. 2020 Jun 3;20:838. doi: 10.1186/s12889-020-08907-y (PMC7268459; doi:10.1186/s12889-020-08907-y)
Supplement: Supplementary file 4 — Additional file 4: Table S1. Key words used according to the bibliographic databases. Table S2. Structured search strategy according to PICOS axiom and bibliographic databases. Table S3. Studies included in the metanalyses for prevalence active tuberculosis (n = 10). Table S4. Studies included in the metanalyses for prevalence of latent tuberculosis infection (n = 13). Chart S1. Prisma Checklist [file 12889_2020_8907_MOESM4_ESM.docx]

**SUPPLEMENT MATERIAL**

**Table S1 – Key words used according to the bibliographic databases**

| **Bibliographic Dataases** | **Key words** |
| --- | --- |
| Medline  EMBASE  Web of Science | *(tuberculos* AND (prevalence OR incidence)) AND (refugee* OR asylum seek* OR forced migrat*)* |
| LILACS | *tuberculos* AND refug** |

*Truncation

**Table S2 – Structured search strategy according to PICOS axiom and bibliographic databases**

| **Strategy** | ***Medline, EMBASE* e *Web of Science*** | **LILACS** |
| --- | --- | --- |
| *Patient* | *Refugee* | Refugiado |
|  | *Asylum seekers* |  |
|  | *Forced migration* |  |
| *Intervention* | *NA* | NA |
| *Comparison* | *NA* | NA |
| *Outcome* | *Incidence* | Tuberculose |
|  | *Prevalence* |  |
|  | *Tuberculosis* |  |
| *Study design* | * | * |

* There were no restrictions in the search regarding the study design.

**Table S3 – Studies included in the metanalyses for prevalence of active tuberculosis (n=10)**

| **Destination Continent** | **Country of origin** | **Number of studies** | **Sample size (n)** | |
| --- | --- | --- | --- | --- |
|  |  |  | **Minimum** | **Maximum** |
| Asia | Afghanistan | 1 | - | 23,152 |
|  | Iraq | 1 | - | 14,077 |
|  | North Korea | 1 | - | 7,722 |
| Europe | Afghanistan | 2 | 166 | 1,996 |
|  | Eritrea | 2 | 83 | 117 |
|  | Ethiopia | 1 | - | 88 |
|  | Gambia | 1 | - | 139 |
|  | Ghana | 1 | - | 34 |
|  | Iraq | 1 | - | 224 |
|  | Nigeria | 1 | - | 523 |
|  | Russia | 1 | - | 182 |
|  | Serbia and Montenegro | 1 | - | 149 |
|  | Somalia | 3 | 26 | 457 |
|  | Syria | 1 | - | 44 |
|  | Tunisia | 1 | - | 32 |
| Americas | Bosnia | 1 | - | 1,711 |
|  | Thailand | 1 | - | 15,455 |
|  | Tibet | 1 | - | 180 |

**Table S4 – Studies included in the metanalyses for prevalence of latent tuberculosis infection (n=13)**

| **Destination Continent** | **Country of origin** | **Number of studies** | **Sample size (n)** | |
| --- | --- | --- | --- | --- |
|  |  |  | **Minimum** | **Maximum** |
| America | Cuba | 1 | - | 241 |
|  | Yugoslavia | 1 | - | 402 |
|  | Iraq | 1 | - | 4,923 |
|  | Tibet | 1 | - | 163 |
|  | Russia | 1 | - | 1,131 |
|  | Somalia | 2 | 149 | 291 |
|  | Thailand | 1 | - | 5,637 |
| Europe | Afghanistan | 2 | 132 | 1,996 |
|  | Eritrea | 2 | 83 | 177 |
|  | Gambia | 1 | - | 139 |
|  | Ethiopia | 1 | - | 88 |
|  | Ghana | 1 | - | 34 |
|  | Nigeria | 2 | 39 | 523 |
|  | Syria | 1 | - | 44 |
|  | Somalia | 2 | 26 | 199 |
|  | Tunisia | 1 | - | 32 |
| Asia | Iraq | 1 | - | 13,669 |
|  | North Korea | 1 | - | 1,112 |

**Chart S1 – Prisma Checklist**

| **Section/topic** | **#** | | **Checklist item** | | **Reported on page #** | |
| --- | --- | --- | --- | --- | --- | --- |
| **TITLE** | | | | |  | |
| Title | 1 | | Identify the report as a systematic review, meta-analysis, or both. | | Title | |
| **ABSTRACT** | | | | |  | |
| Structured summary | 2 | | Provide a structured summary including, as applicable: background; objectives; data sources; study eligibility criteria, participants, and interventions; study appraisal and synthesis methods; results; limitations; conclusions and implications of key findings; systematic review registration number. | | Abstract | |
| **INTRODUCTION** | | | | |  | |
| Rationale | 3 | | Describe the rationale for the review in the context of what is already known. | | Introduction, paragraph 5 | |
| Objectives | 4 | | Provide an explicit statement of questions being addressed with reference to participants, interventions, comparisons, outcomes, and study design (PICOS). | | Introduction, paragraph 5 | |
| **METHODS** | | | | |  | |
| Protocol and registration | 5 | | Indicate if a review protocol exists, if and where it can be accessed (e.g., Web address), and, if available, provide registration information including registration number. | | Methods, paragraph 11 | |
| Eligibility criteria | 6 | | Specify study characteristics (e.g., PICOS, length of follow-up) and report characteristics (e.g., years considered, language, publication status) used as criteria for eligibility, giving rationale. | | Methods, paragraph 2, 4, 5 and 6 | |
| Information sources | 7 | | Describe all information sources (e.g., databases with dates of coverage, contact with study authors to identify additional studies) in the search and date last searched. | | Methods, paragraph 1 and 2 | |
| Search | 8 | | Present full electronic search strategy for at least one database, including any limits used, such that it could be repeated. | | Supplement | |
| Study selection | 9 | | State the process for selecting studies (i.e., screening, eligibility, included in systematic review, and, if applicable, included in the meta-analysis). | | Methods, paragraph 3 | |
| Data collection process | 10 | | Describe method of data extraction from reports (e.g., piloted forms, independently, in duplicate) and any processes for obtaining and confirming data from investigators. | | Methods, paragraph 7 | |
| Data items | 11 | | List and define all variables for which data were sought (e.g., PICOS, funding sources) and any assumptions and simplifications made. | | Methods, paragraph 10 | |
| Risk of bias in individual studies | 12 | | Describe methods used for assessing risk of bias of individual studies (including specification of whether this was done at the study or outcome level), and how this information is to be used in any data synthesis. | | NA | |
| Summary measures | 13 | | State the principal summary measures (e.g., risk ratio, difference in means). | | Methods, paragraph 9 and 10 | |
| Synthesis of results | 14 | | Describe the methods of handling data and combining results of studies, if done, including measures of consistency (e.g., I^2^) for each meta-analysis. | | Methods, paragraph 10 | |
| Risk of bias across studies | | 15 | | Specify any assessment of risk of bias that may affect the cumulative evidence (e.g., publication bias, selective reporting within studies). | | Methods, paragraph 8 |
| Additional analyses | | 16 | | Describe methods of additional analyses (e.g., sensitivity or subgroup analyses, meta-regression), if done, indicating which were pre-specified. | | Methods, paragraph 10 |
| **RESULTS** | | | | | |  |
| Study selection | | 17 | | Give numbers of studies screened, assessed for eligibility, and included in the review, with reasons for exclusions at each stage, ideally with a flow diagram. | | Results, paragraph 1 and 2 |
| Study characteristics | | 18 | | For each study, present characteristics for which data were extracted (e.g., study size, PICOS, follow-up period) and provide the citations. | | Results, “Study Characteristics and Population” |
| Risk of bias within studies | | 19 | | Present data on risk of bias of each study and, if available, any outcome level assessment (see item 12). | | Results, “Risk of bias” |
| Results of individual studies | | 20 | | For all outcomes considered (benefits or harms), present, for each study: (a) simple summary data for each intervention group (b) effect estimates and confidence intervals, ideally with a forest plot. | | NA |
| Synthesis of results | | 21 | | Present results of each meta-analysis done, including confidence intervals and measures of consistency. | | Results, “Main findings” |
| Risk of bias across studies | | 22 | | Present results of any assessment of risk of bias across studies (see Item 15). | | Results, “Risk of bias” |
| Additional analysis | | 23 | | Give results of additional analyses, if done (e.g., sensitivity or subgroup analyses, meta-regression [see Item 16]). | | Results, “Main findings” – Subgroup analyses |
| **DISCUSSION** | | | | | |  |
| Summary of evidence | | 24 | | Summarize the main findings including the strength of evidence for each main outcome; consider their relevance to key groups (e.g., healthcare providers, users, and policy makers). | | Discussion, paragraph 1 |
| Limitations | | 25 | | Discuss limitations at study and outcome level (e.g., risk of bias), and at review-level (e.g., incomplete retrieval of identified research, reporting bias). | | Discussion, paragraph 5 and 6 |
| Conclusions | | 26 | | Provide a general interpretation of the results in the context of other evidence, and implications for future research. | | Conclusion |
| **FUNDING** | | | | | |  |
| Funding | | 27 | | Describe sources of funding for the systematic review and other support (e.g., supply of data); role of funders for the systematic review. | | Funding |
